# Exceptional Use of Sex Pheromones by Parasitoids of the Genus *Cotesia*: Males Are Strongly Attracted to Virgin Females, but Are No Longer Attracted to or Even Repelled by Mated Females

**DOI:** 10.3390/insects5030499

**Published:** 2014-06-30

**Authors:** Hao Xu, Nathalie Veyrat, Thomas Degen, Ted C. J. Turlings

**Affiliations:** Laboratory of Fundamental and Applied Research in Chemical Ecology (FARCE), Institute of Biology, University of Neuchâtel, CH-2000 Neuchâtel, Switzerland; E-Mails: hao.xu@unine.ch (H.X.); nathalie.veyrat@unine.ch (N.V.); thomas.degen@unine.ch (T.D.)

**Keywords:** parasitoids, mate finding strategy, sex pheromones, repellency, *Cotesia glomerata*, *Cotesia marginiventris*, gregarious, solitary

## Abstract

Sex pheromones have rarely been studied in parasitoids, and it remains largely unknown how male and female parasitoids locate each other. We investigated possible attraction (and repellency) between the sexes of two braconid wasps belonging to the same genus, the gregarious parasitoid, *Cotesia glomerata* (L.), and the solitary parasitoid, *Cotesia marginiventris* (Cresson). Males of both species were strongly attracted to conspecific virgin females. Interestingly, in *C. glomerata*, the males were repelled by mated females, as well as by males of their own species. This repellency of mated females was only evident hours after mating, implying a change in pheromone composition. Males of *C. marginiventris* were also no longer attracted, but not repelled, by mated females. Females of both species showed no attraction to the odors of conspecific individuals, male or female, and *C. glomerata* females even appeared to be repelled by mated males. Moreover, the pheromones were found to be highly specific, as males were not attracted by females of the other species. Males of *Cotesia glomerata* even avoided the pheromones of female *Cotesia marginiventris*, indicating the recognition of non-conspecific pheromones. We discuss these unique responses in the context of optimal mate finding strategies in parasitoids.

## 1. Introduction

The reproductive success of parasitoids is tightly linked to the females’ ability to find hosts for their offspring, but optimal foraging for food and mates is also essential to maximize adult lifespan and fitness. Having a haplo-diploid sex determination system, female hymenopteran parasitoids are able to reproduce without mating, but this will result in only male progeny. Hence, newly emerged females of parasitic wasps are facing the trade-off decision of either locating hosts as soon as possible, producing only male offspring, or first investing time and energy into mating before ovipositing, as this would allow them to produce both males and females. Generally, the latter option is observed to be favored [1,2,3,4]. Therefore, an effective mate-finding strategy can be expected in parasitoids. Indeed, the relatively few studies that have investigated mate finding in parasitoids have found evidence for sex pheromones (e.g., [5,6,7,8]). Mate finding in parasitoids is mainly based on pheromones released by females, although on some occasions, the roles are reversed, with the males also releasing sex pheromones [9,10]. After mating, females switch from releasing pheromones or searching for males to searching for hosts [3,11,12]. This switch may be immediate or take more than 24 hours after mating [3,11,13]. For example, mated females of *Nasonia vitripennis* stop responding to the pheromones released by males just five minutes after mating [8]. Mated females of *Cotesia vestalis* are strongly attracted by host‑induced plant volatiles, to which virgin females, by contrast, are indifferent [12]. The fact that mated females cease to release pheromones gives other, still virgin females more opportunities to attract mates, and the mated females are less harassed by males and, therefore, can better concentrate on host location [8].

Gregarious parasitoids, when they leave their host, usually clump their cocoons together, and wasps show synchronized emergence as adults. This means that the broods are normally subject to local mate competition and probably inbreeding [9,14]. By comparison, the offspring of the solitary parasitoids are more widely dispersed and face an entirely different challenge to find suitable mates. These differences may have resulted in distinct mate finding strategies. It is likely that mated females of gregarious parasitoids may be readily disturbed by the nearby males, whereas solitary parasitoids are more likely to find a host without encountering other males. Little is known about possible differences in mating behaviors between gregarious and solitary parasitoids.

Only a few studies have dealt with the use of pheromones by parasitic wasps, especially in the context of specificity. For the genus, *Melittobia*, it is known that males of certain species are also attractive to females of closely-related species [15]. The braconids, *Cotesia flavipes, Cotesia sesamiae* and *Cotesia chilonis*, all larval parasitoids with stem-boring lepidopteran hosts, mate interspecifically, and in olfactometer assays, males of *C. sesamiae* have been found to be slightly attracted to virgin *C. chilonis* females [16]. The pteromalid pupal parasitoid, *Trichomalopsis sarcophagae*, shares the same pheromone components with members of the sister genus, *Nasonia*, and in the case of *N. vitripennis*, specificity is assured through the production of an additional stereoisomer component [17].

*Cotesia* is a very species-rich genus in the Braconidae (Hymenoptera), with an estimated number of nearly one thousand species distributed worldwide [18]. The group is significant for both practical applications in biological control and fundamental ecological research [18]. Females normally mate once, while males can mate several times throughout their life [16,19]. Given the species richness, it is surprising that studies on the pheromones in the group are so rare. What is known is, for example, that both sexes of *C. flavipes* attract each other by volatile and contact pheromones [16,19], and females of *C. rubecula* attract males with air-borne chemicals [20]. By contrast, mate finding in *C. sesamiae* and *C. chilonis* is not based on the release of volatile pheromones [16]. In *C. glomerata*, a model species for parasitoid research, there is some evidence from a field study that sex pheromones are not only produced by mature females, as males are already attracted before the females emerge from their cocoons [21,22]. Another well-studied *Cotesia* species is the generalist *C. marginiventris*, which is frequently used as an inundative biological control agent, but its pheromones and mating behaviors are as yet unexplored. In this study, we studied the mate finding behavior of gregarious *C. glomerata* and solitary *C. marginiventris* using a series of six-arm olfactometer assays. Thus, we obtained detailed information on the conspecific and heterospecific attraction for both sexes. 

## 2. Experimental Section

### 2.1. Wasps

The two endoparasitoids were reared in our laboratory at the University of Neuchatel. The *C. glomerata* rearing was started with individuals that had emerged from *Pieris brassicae* caterpillars collected from cabbage plants grown in gardens around Neuchatel, Switzerland. The offspring was reared on *P. brassicae* (first–second instar) that was fed on cabbage. *C. marginiventris* were initially obtained from the United States Department of Agriculture-Agricultural Research Service (USDA‑ARS), Biological Control and Mass Rearing Research Unit (Stoneville, MI, USA), occasionally replenished with individuals from field collections in Mexico and reared as described in Tamò *et al.* [23]. The hosts were about three-day old *Spodoptera littoralis* caterpillars (first–second instars), which were fed with a wheat germ-based artificial diet. The eggs of *S. littoralis* were provided by Syngenta (Stein, Switzerland). To obtain virgin adult wasps, each parasitoid cocoon was placed in a 1.5-mL centrifuge tube until a wasp emerged. Then, virgin females and males were kept separately in two Bugdorm-1 cages (30 × 30 × 30 cm, Mega View Science Education Services Co. Ltd, Taiwan), provided with honey and moist cotton wool in a 25 °C incubator (LD 16:8 h) for three days before each test. To obtain mated individuals, one female and one male were placed in a Petri dish (90 × 15 mm) and then tested immediately or stored in Bugdorm-1 cages with honey and moist cotton wool in a 25 °C incubator for about 18 or 40 hours before tests.

### 2.2. Bioassay

**Figure 1:**
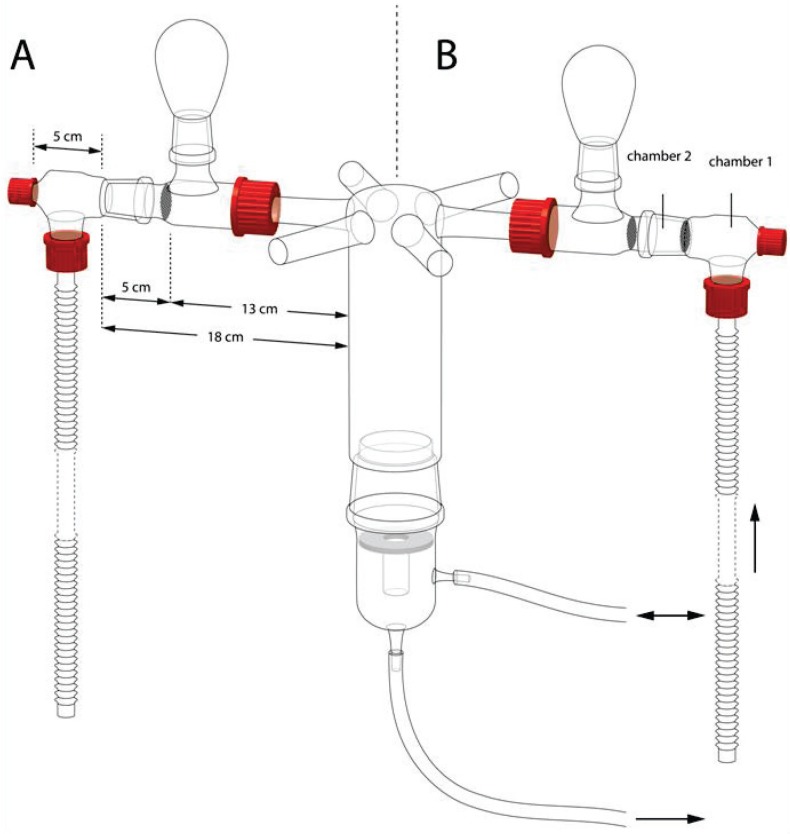
The six-arm olfactometer as it was used to test for attractiveness (**A**) and repellency (**B**). For the repellency tests, Chamber 1 contained virgin females (the source of attraction) and Chamber 2 either no wasps (control) or mated females or males (the source of potential deterrents).

The bioassays were carried out in a six-arm olfactometer, as described by Turlings *et al.* [24]. Each arm had an air flow of 0.6 L per minute that entered the central release chamber (Figure 1). Six virgin wasps were released at the same time in this central chamber, where they were allowed to choose among the arms within 30 minutes. From previous studies (*i.e.*, [23,24]), we know that the wasps are initially attracted to the light above the olfactometer. When attracted by an odor, they will enter the respective arm and walk until they encounter metal screens that prevent them from walking further into the arm. At this point, they move upwards into a glass bulb that is located just before the metal screen (Figure 1). They will readily stay in these bulbs for the duration of the experiment. Thirty minutes after their release, the wasps in the bulbs were counted and removed, before a new group of six wasps was released. 

Test for the presence of female pheromones: Six virgin females, six newly mated females and six unmated males were placed into three non-adjacent arms, respectively, and the three control arms in between were left empty. Several overlapping layers of metal meshes kept the “treatment” wasps from entering the central chamber, prevented the choosing wasps from approaching the pheromone source and reduced any potential visible cues (Figure 1A). Unmated males were released in the central chamber. The test was repeated four times, with six releases of six unmated males (4 × 6 × 6) on each experimental day, for a total of 144 wasps per experiment. Between replicates, the positions of the different treatments in the setup were changed.

Test for the presence of male pheromones: Six unmated males, six newly mated males and six virgin females were placed as odor sources in the olfactometer with the same design as above. There were four releases of six virgin females per replicate. This assay was replicated four times, with a total of 96 wasps.

Test for repellency of mated females: To test if the observed poor responses to already mated females in the previous experiments were due to repellency, we combined odor sources as follows. Six virgin females were combined with six mated females; six virgin females were combined with six unmated males; and six virgin females were kept alone in each of the three non-adjacent arms separated by empty arms (control). The combined groups of six wasps were placed in two chambers in the olfactometer, as shown in Figure 1B: Chamber 1 contained the virgin females, and Chamber 2 included the six mated females (0, 18 or 40 hours after mating), or six unmated males, respectively, or was left empty. Each test was replicated four times with new wasps as odor sources and 6 × 6 unmated males each time (144 wasps).

Test for species specificity of *Cotesia* pheromones: Six virgin females of *C. glomerata* and *C. marginiventris* were placed into two opposite arms, and the remaining four arms were left empty. Unmated males (6 × 6) of either species were released into the central chamber to test their responses to these odor sources. An additional test was done to assess the responses to the interspecific pheromone in the absence of the conspecific pheromone. For this, only one arm of the olfactometer contained six virgin *C. glomerata* or *C. marginiventris* females, while the other five arms were left empty. Unmated males of the other species (6 × 6) were released into the central chamber. Each test was repeated four times (144 wasps).

Statistics: To test whether the differences among the responses of the parasitoids to the treatments were significant, we used generalized linear models (GLMs) with the assumption that the arms are equally chosen by parasitoids without stimuli [24]. The models take into account the possible effects of over-dispersion caused, for instance, by positional biases or wasps affecting each other’s responses [25]. Each model was fitted by maximum quasi-likelihood estimation in the software package, R. In the figures, the number of wasps choosing empty arms was divided by the number of empty arms present in the setup to make it comparable to the other treatments. 

## 3. Results

**Figure 2:**
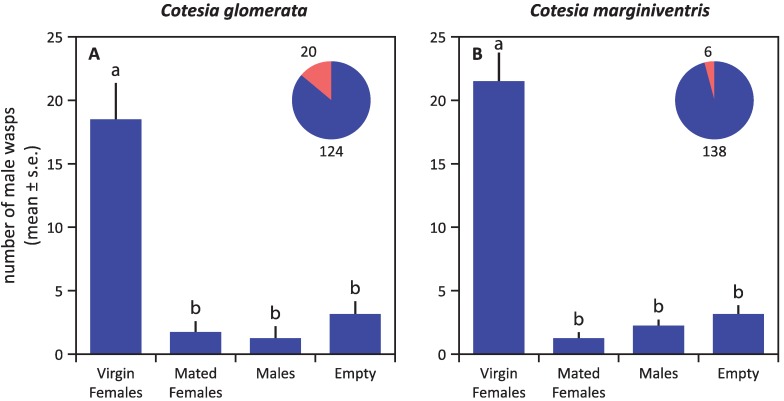
Responses of unmated males to conspecific virgin females, mated females and males in *C. glomerata* (**A**) and *C. marginiventris* (**B**) in a six-arm olfactometer*.* The proportion of wasps that showed no response, *i.e.*, that did not choose an arm, is given in red in the pie chart, supplemented with the absolute numbers of wasps. Statistical differences (*p* < 0.05) are indicated with different letters above the bars.

**Figure 3:**
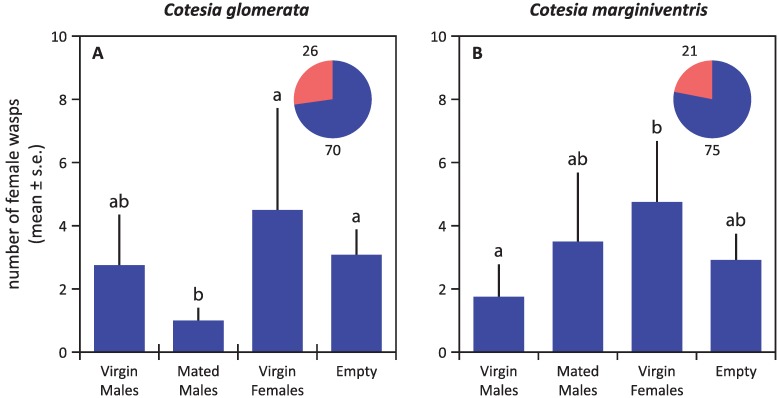
Responses of virgin females to conspecific virgin males, mated males and virgin females in *C. glomerata* (**A**) and *C. marginiventris* (**B**) in a six-arm olfactometer*.* The proportion of wasps that showed no response, *i.e.*, that did not choose an arm, is given in red in the pie chart, supplemented with the absolute numbers of wasps. Statistical differences (*p* < 0.05) are indicated with different letters above the bars.

In both *Cotesia* species, virgin females were strongly attractive to males, whereas olfactometer arms containing mated females or males did not differ significantly from empty control arms in the number of male wasps that entered (Figure 2). Virgin females were not attracted to conspecific males (irrespective of mating status) or to virgin females (Figure 3). The overall pattern of responses was very similar for both species, except for the fact that virgin females of *C. glomerata* seem to avoid the arm containing mated males, which was not the case for *C. marginiventris* (Figure 3A).

**Figure 4:**
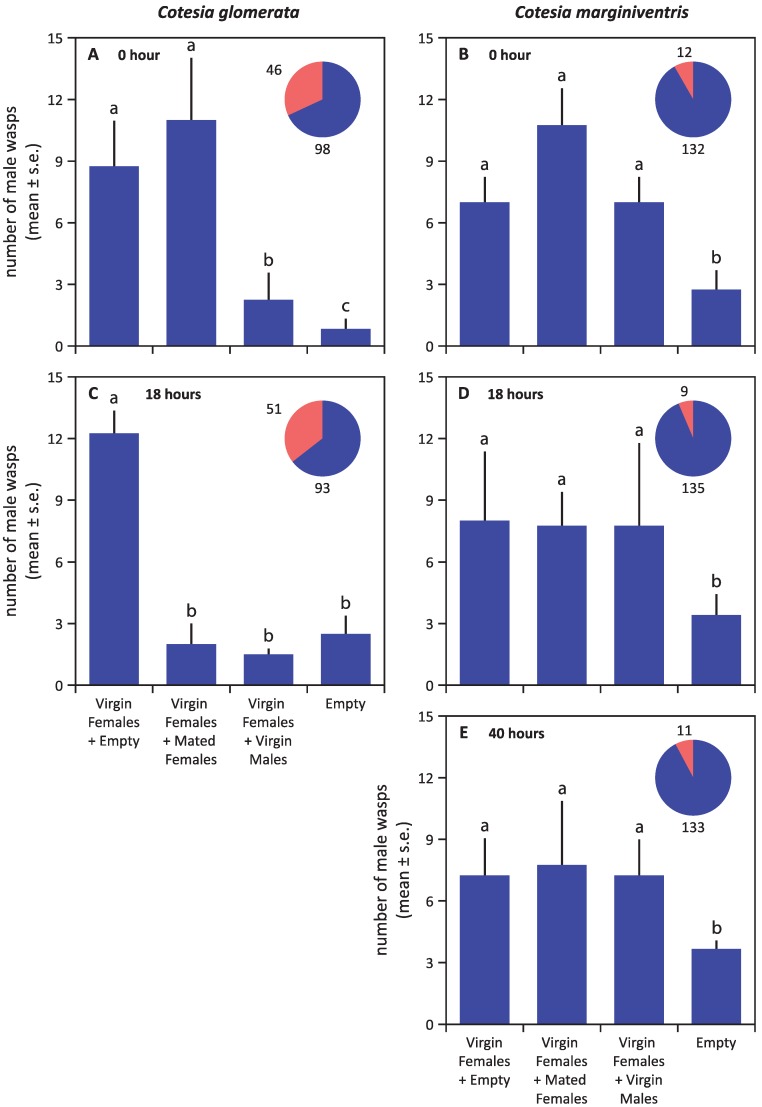
The six-arm olfactometer test for the repellency of mated females and of unmated males in the two parasitoid species, *C. glomerata* (**A**,**C**) and *C. marginiventris* (**B**,**D**,**E**), immediately (**A**,**B**), about 18 hours (**C**,**D**) and about 40 hours (**E**) after females had mated, respectively. The proportion of wasps that showed no response, *i.e.*, that did not choose an arm, is given in red in the pie chart, supplemented with the absolute numbers of wasps. Statistical differences (*p* < 0.05) are indicated with different letters above the bars.

After mating, females lost their attractiveness to males (Figure 2 and Figure 4). In *C. glomerata*, mated females were even strongly repellent to the males several hours after mating (Figure 4C). No such repellency was found for *C. marginiventris* (Figure 4D,E). Virgin males were also repellent to males themselves in *C. glomerata*, but not in *C. marginiventris* (Figure 4).

**Figure 5:**
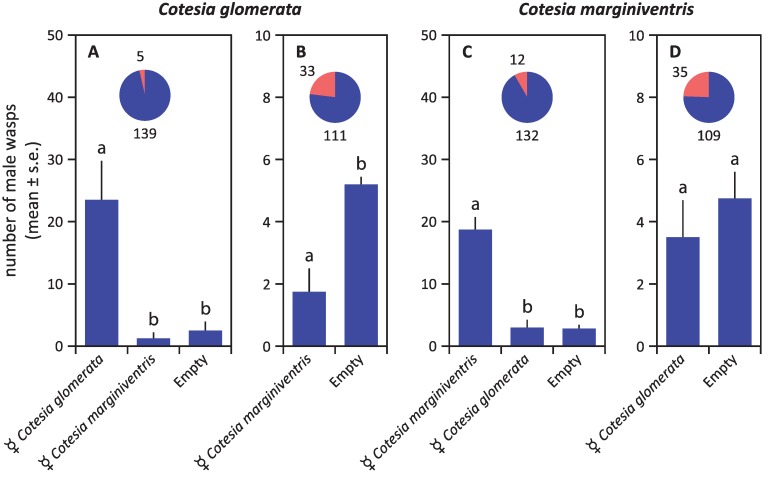
Cross-attractant responses of male wasps, *C. glomerata* (**A**,**B**) and *C. marginiventris* (**C**, **D**), to con- and inter-specific female cues in a six-arm olfactometer. Interspecific cues were either tested in combination with conspecific cues (**A**,**C**) or alone (**B**,**D**). The proportion of wasps that showed no response, *i.e.*, that did not choose an arm, is given in red in the pie chart, supplemented with the absolute numbers of wasps. Statistical differences (*p* < 0.05) are indicated with different letters above the bars.

In the cross-attraction tests, males of *C. glomerata* and *C. marginiventris* were not attracted by females of the other species, indicating that the pheromones are highly specific (Figure 5). Males of *C. glomerata* even avoided the odor of *C. marginiventris* females (Figure 5B), whereas males of *C.* *marginiventris* did not respond to female *C. glomerata* (Figure 5D).

## 4. Discussion

### 4.1. Mate Finding Strategy of Cotesia

Our study reveals that virgin *Cotesia* females attract conspecific males by volatizing pheromones. In the two studied species, virgin males do not attract females from a distance (about 15 cm in our test, Figure 1A). As a gregarious parasitoid, it should be easier for males of *C. glomerata* to find females to mate as compared to the solitary sister species. We frequently observed that males of *C. glomerata* emerged earlier and stayed on or near the cocoon cluster, waiting for the females to emerge. The attractant has been shown to be emitted already at the late developmental “black-eye” stage of the cocoons [21]. Mating normally takes place soon after female emergence within 10 cm from the natal patch, which is within the pheromonal functional range [22]. In our tests, the odor cues were attractive at a distance of at least 18 cm (Figure 1B). Not all individuals mate on the natal patch: the percentage of *C. glomerata* wasps mating on the natal patch has been estimated at about 60% [22]. Since there are very few females that parasitize their hosts without mating in the field [1], outbreeding must be considered an important part of the mating strategy of *C. glomerata*. However, the pheromones are apparently active only over relatively short distances. We found that the attraction over a distance of 18 cm (Figure 4A,C) was lower compared to a 13 cm distance (Figure 2A), as indicated by the response rate. Response rates are further reduced at a greater distance [26]. Over longer distances, the mating strategies of these and other parasitoids may also involve host‑induced plant volatiles (HIPVs). *Cotesia plutellae*, for instance, probably uses both sex pheromones and plant volatiles for mate finding at a long distance [27], and a few studies suggest that HIPVs aid in the mate finding in other genera and families of hymenopteran parasitoids [28,29], but direct evidence is still lacking. *C. glomerata* is also able to use visual cues to locate mates [30]. 

Compared with the gregarious parasitoids, for solitary wasps, the challenge to find a mate would logically require detection over longer distances. Yet, certain solitary wasps may mate in their natal patch, because the hosts are aggregated, as is the case for aphids and scale insects [31]. This is not the case for caterpillars that serve as hosts for *C. marginiventris*, which generally disperse after hatching [32,33,34]. Therefore the offspring hatching from these hosts has to engage in a longer-range mate search. Our results imply that males actively search for females and that they use a pheromone released by females.

*Cotesia* females mate only once [16,19]. After mating, the female parasitoids switch their foraging behavior to host finding. Indeed, we found that mated females of the two *Cotesia* species lost their attractiveness rapidly after mating (Figure 2), and mated females of *C. glomerata* even became repellent to males (Figure 4). Something similar has been reported for the braconid aphid parasitoids, *Aphidius nigripes* and *Aphidius ervi*: in a field study, mated females attracted significantly fewer males than did virgin females [5,7]. Secretions transferred during male ejaculation may inhibit pheromone production in females. This phenomenon is known as pheromonostasis [35,36] and is known to occur in dipterans and lepidopterans [11,13,37]. For certain Lepidoptera, including *Pieris* species that serve as hosts of *C. glomerata*, it has even been found that males transfer so-called “anti-aphrodisiacs” to females, resulting in reduced attraction and even repellency [38,39]. This in turn may attract egg parasitoids in search of a ride to oviposition sites [40,41]. An analogous phenomenon has been reported for *Drosophila melanogaster*, where cuticular hydrocarbons that are characteristic for males have anti-aphrodisiac properties and are transferred to females during mating [42,43]. Several hours later, mated females even appear to mimic males by synthesizing this same compound to deter courting males [42]. It is possible that the transfer of “anti-aphrodisiacs” also occurs in the studied *Cotesia* wasps. In *C. glomerata*, it could explain why mated females, as well as males are repellent to conspecific males, but the fact that the females are not yet repellent right after mating (Figure 4) suggests a change that occurs in the females. Alternatively, the males might deposit a repellent pheromone during copulation that needs some time to be released or activated. 

### 4.2. Possible Differences in Mating Behaviors between Gregarious and Solitary Parasitoids

Due to their distinct distribution within a colony, gregarious and solitary parasitoids can be expected to differ in their pheromone communication system. For *C. glomerata*, mated females were found repellent to males. This allows males to optimize their efforts to find unmated females and minimizes disturbance of females by males while they forage for hosts. In contrast, mated females of *C. marginiventris* exert no repellent effect upon males (Figure 4).

Parasitoids are expected to choose the lower-cost option between actively searching for mates and the biosynthesis of pheromones in sexual communication [10]. It is not likely for both sexes to release the pheromones at the same range. When the individuals hatch close to each other, as is the case with gregarious parasitoids, it could be expected that the wasps do not invest in costly pheromone release. This is clearly not the case for *C. glomerata*, since the females emit pheromones in spite of the large proportion of females that mate in the natal patch [1,22]. The attractiveness of the pheromones was at least as strong in *C. glomerata* as in *C. marginiventris*, suggesting that at this level, there is no difference in the investment in pheromones between the solitary and gregarious parasitoid.

*Cotesia* females mate only once, but the males are able to mate several times during their lifetime [16,19]. Interestingly, females of *C. glomerata* avoided mated males in the six-arm olfactometer (Figure 3A). Apparently, females give a priority to unmated males for mating, possibly because they would transfer more sperm. In addition, males of *C. glomerata* are repelled by conspecific males. Males emerge earlier than females, and then, most of them stay around the clustered cocoons, fanning their wings or waiting motionless on an unemerged cocoon [44]. Therefore, the observed mutual repellency may promote male dispersal and reduce competition. Considering that most males in a patch will be 100% related, this competition avoidance will be highly adaptive. The male-male repellency was not observed in *C. marginiventris*, where competition among brothers is much less evident, as their cocoons are much more widely distributed.

### 4.3. Species Specificity of Sex Pheromones

In several parasitoids, some cross-attraction between related species has been observed [15,16,17]. In contrast, we found the specificity of pheromonal responses in the two *Cotesia* species to be very high. *C. glomerata* males even avoided the odor of *C. marginiventris* females (Figure 5B). To our knowledge, no evidence of repellency between different parasitoid species has previously been reported. The apparent recognition between the two *Cotesia* species indicates a close evolutionary relationship and enforces prezygotic reproductive isolation. The genus, *Cotesia*, is exceedingly diverse [18], and pheromone-mediated avoidance among different genotypes may have contributed to this diversity. The identification of the actual pheromones that are involved in the interactions is necessary to further analyze the relationships among *Cotesia* species.

## 5. Conclusion

The sexual communication system in the two *Cotesia* species was found to rely on pheromones released by the females. There was no indication that females use male pheromones for mate finding, but it is not excluded that, upon contact, male-produced pheromones play a role in mate choice. Males of the two tested parasitoids showed very similar and highly specific pheromonal responses. The only difference was that males of the gregarious parasitoid were repelled by mated females and males, whereas the solitary parasitoid showed no response to these sources. The observed repellencies in the gregarious parasitoid may be linked to the initial high aggregation of the offspring around the natal patch. Once they have mated, aggregation is no longer adaptive, and repellency may facilitate proper dispersal and help to optimize foraging for a host. 
